# Investigations on the Effects of a Passive Standing-from-Squatting and Gait Assistive Exoskeleton on Human Motion

**DOI:** 10.3390/bioengineering12060590

**Published:** 2025-05-30

**Authors:** Yu-Chih Lin, Sih-You Lin, Shih-Yu Kao

**Affiliations:** Department of Mechanical and Mechatronic Engineering, National Taiwan Ocean University, No.2, Beining Rd., Jhongjheng District, Keelung 20224, Taiwan; you1525@yahoo.com (S.-Y.L.); iclgary5555@gmail.com (S.-Y.K.)

**Keywords:** exoskeleton, electromyography, gait analysis, squatting

## Abstract

The aim of this study is to examine the biomechanical interaction between an assistive wearable exoskeleton and the human body. For this purpose, a passive exoskeleton is designed to provide support during the transition from a squatting position to standing, while also enabling the resilient components to become active during the initial and mid-swing phases of level walking. The active period can be adjusted by a slot, which triggers the activation of the resilient components when the exoskeleton’s flexion angle exceeds a critical value. This study also compares the effect of using different passive powered components in the exoskeleton. Electromyography (EMG) signals and angular velocity during human motion are collected and analyzed. Experimental results indicate that the designed assistive exoskeleton effectively reduces muscle effort during squatting/standing motion, as intended. The exoskeleton reduces the flexion/extension (*x*-axis) angular velocity during both squatting/standing and the swing phase of gait. The oscillation of the angular velocity curve about the *y*-axis during gait is larger without the exoskeleton, suggesting that the exoskeleton may introduce interference but also a stabilizing effect in certain dimensions during gait. This study provides a stronger foundation for advancing the design of both passive and active powered exoskeletons.

## 1. Introduction

Exoskeletons have the capability to combine a human’s inherent walking adaptability with the intelligence and endurance of a machine. They provide several advantages, including assisting individuals in walking on uneven terrain and carrying heavy loads for military, emergency, and medical purposes. This is achieved by applying assistive torques to the joints through the use of actuators or resilient components, which amplify human muscle strength. Compared to wheeled vehicles, exoskeletons offer superior ability and a broader scope of applications.

The exoskeleton’s very early design was completed by Yagn in 1890 [1] for walking, running, and jumping assistance. It has no external power source but is activated by the deformation energy stored in the bow spring during walking. The first exoskeleton designed with power was a full-body mechanism weighing 680 kg. The exoskeleton, named “Hardiman” of the General Electric (GE) project, consists of 30 servo-controlled joints which are powered by hydraulic components [2]. Zoss et al. [3] designed an exoskeleton with seven degrees of freedom in each leg, named the Berkeley Lower Extremity Exoskeleton (BLEEX). They can be used for military or emergency rescue purposes due to their load-carrying capacity in a wide range of terrains, including bumpy or uneven terrain and steps. Apart from labor assistance [4], exoskeletons are also developed for the rehabilitation of patients with injuries of the nervous or musculoskeletal systems and to provide motion assistance for individuals with mobility impairments [5,6,7,8,9]. The development and application of lower limb exoskeletons for walking and running are reviewed [10,11]. They highlighted that exoskeletons can be utilized not only for individuals with disabilities but also for non-disabled individuals, aiming to enhance industrial or military performance. The current limitations of exoskeletons are also addressed, which include their heavy weight, limited torque and power, the continued reliance on crutches for paralyzed patients, high costs, alterations to the biomechanics of human gait resulting in increased metabolic cost, noise generation, and difficulties in information exchange between the wearer and the exoskeleton system.

In order to reduce the weight and minimize unnatural active motion of the exoskeleton, passive or quasi-passive mechanisms are designed [12,13,14,15]. The resilient components are used instead of heavy actuators. The design of the passive or quasi-passive structure can overcome the problems of high costs and the requirement for large power or long-time recharging. However, whether it is an active or passive powered assistive exoskeleton, some designs can reduce effort in certain aspects of physiological measurement data, while others cannot. The increase in effort when wearing the exoskeleton may be caused by the learning effect of users when adapting to the exoskeleton, the increased mass and resulting constraints imposed by the exoskeleton, and the nonlinear relationship between work reduction and muscle activation.

The exoskeleton is also designed to provide squatting assistance [16,17,18,19,20,21,22,23,24]. The researchers have verified that wearable exoskeletons can reduce physical effort and fatigue during squatting. The experimental results show a significant reduction in muscle activity. They also confirmed the effectiveness of the exoskeleton’s balancing and supporting assistance functions. Given that the exoskeleton is a device worn on the human body, the interaction between the device and the body is of paramount importance in the design process. The exoskeletons with uniaxial rotation joints may not accurately replicate the complex motion of real biological joints [16]. Researchers have also developed various control strategies to make the exoskeleton’s movements more closely resemble human motion [25,26].

However, the development of exoskeletons still requires a deeper understanding and further improvements in efficiency, as the factors affecting walking efficiency may seem simple but are actually complex [14]. While the exoskeleton can provide motion power and supporting force for the joints, it is crucial to consider its potential impact on hindering limb motion. Although certain studies have examined exoskeletons through biomechanical experiments [26,27,28,29,30,31,32], there is still a lack of comprehensive understanding regarding the interaction between the human body and the exoskeleton from a biomechanical perspective. In light of this, the aim of this study is to analyze the impact of a passive squatting/standing and walking assistive exoskeleton on daily motion, exploring both its interfering and assisting effects, investigating influencing factors, and enhancing design knowledge.

## 2. Methods

An exoskeleton featuring resilient components is designed for standing-from-squatting motion assistance and allowing the resilient components to remain inactive during most durations of level walking, but initial and mid-swing phases when the exoskeleton’s flexion angle exceeds a critical value. Three kinds of resilient components are used: helical compression springs, hydraulic cylinder rods, and torsion springs, which can provide the recovery supporting force when standing after squatting motion and the extension motion in the swing phase of gait. The helical compression spring can provide a supporting force proportional to the compression length, while the torsion spring provides a torque that increases with the torsional angle. The hydraulic cylinder rod is designed not to immediately rebound when subjected to an impact, allowing for a gradual contraction and extension to absorb the impact energy, and is expected to provide a stable supporting force for users during squatting/standing and prevent sudden changing forces that could potentially unbalance elderly or disabled individuals. Through the design of a slot in the mechanism, the resilient component is only compressed when the exoskeleton’s flexion angle exceeds a critical value. In this study, we have set the active region of the resilient component to be during the initial and mid-swing phases of level walking, and the effect of the involvement of the resilient component on walking is studied.

To assess the impact of the exoskeleton, ten healthy subjects, including one female and nine male subjects, are requested to walk on level ground and perform the squatting/standing motion both with and without the exoskeleton. The detailed description is mentioned in section B, “Biomechanical experiment”. The Trigno Wireless Biofeedback System (Delsys Inc., Natick, MA, USA) was used to collect electromyography (EMG) data and angular velocity. Subsequently, the disparities in lower limb motion between wearing and not wearing the exoskeleton were analyzed.

### 2.1. Exoskeleton Design

**Figure 1:**
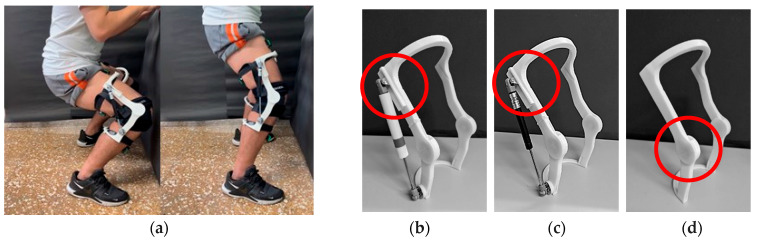
The physical model of the exoskeleton (**a**) The subject wears the exoskeleton (**b**) The exoskeleton with helical compression spring (**c**) The exoskeleton with hydraulic cylinder rod (**d**) The exoskeleton with torsion spring. The red circles in (**b**–**d**) indicate the position of the slot.

**Figure 2:**
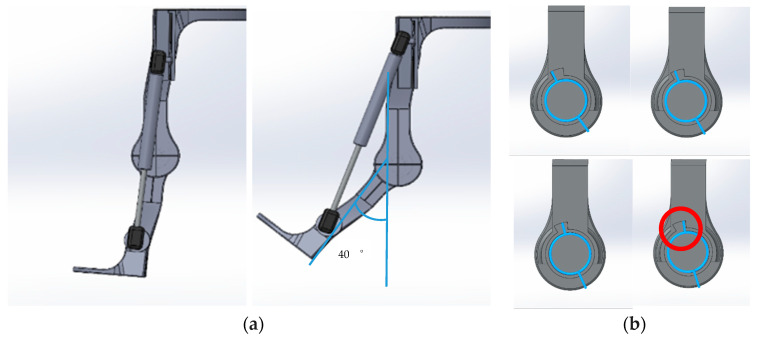
The designed slot on (**a**) a hydraulic cylinder rod and helical compression spring, and (**b**) a torsion spring. The red circle in (**b**) indicates the position where the torsion spring contacts the end of the slot.

**Figure 3:**
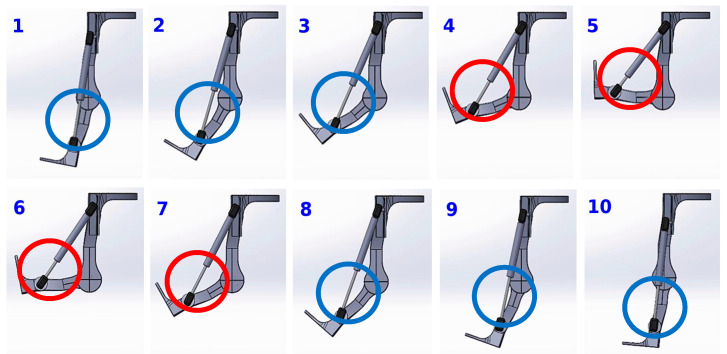
The positions of the exoskeleton at various angles. The figure divides the knee movement from extension to flexion and back to extension into ten steps, as indicated by the numbers 1–10. The circles in each step highlight the exposed length of the resilient component extending from the covering tube. This exposed segment visually represents the compression state of the resilient component. Blue circles indicate the component is in an uncompressed state, while red circles indicate that it is compressed.

A lightweight, passive-powered, assistive exoskeleton with a simple mechanism is designed to eliminate the intricate variables of weights and unnatural active power of actuators. This design aims to clarify the factors influencing body motion while using the exoskeleton. The designed exoskeleton is shown in Figure 1. In order to provide assistance in standing-from-squatting and some specific duration of gait without obstruction by the resilient component in most level walking, a slot is designed for helical compression springs, hydraulic cylinder rods, and torsion springs as shown in the red circles of Figure 1 and the Solidworks design drawings in Figure 2. Through the designed slot on the mechanism, the resilient components will not be compressed until a critical value of the flexion angle of the thigh and shank component. In this experiment, the critical angle is set to be 40 degrees for the exoskeleton. The resilient component will not be compressed during most of the normal speed level walking motion. Once the exoskeleton’s flexion angle surpasses 40 degrees, the resilient component will undergo compression. Except for squatting, this compressed state primarily occurs during the initial and mid-swing phases of normal-speed level walking. Additionally, the extent of unrestricted movement can be fine-tuned by modifying the slot dimensions based on requirements. Figure 3 shows the positions of the exoskeleton at various angles. During steps 1–2, the knee begins to bend, but the critical angle has not yet been reached. The upper end of the resilient component has not contacted the top of the slot, so the resilient component remains uncompressed. As observed in the blue-circled area, there is no change in the length of the resilient component. At step 3, the upper end of the resilient component contacts the top of the slot. As the knee continues to bend, the resilient component becomes compressed. This can be observed in steps 4–5, where the red-circled area shows a shortening of the resilient component. Following this, the knee begins to extend. However, during steps 6–7, the resilient component remains compressed, thus capable of providing a rebound force. As knee extension continues, by step 8, the upper end of the resilient component starts to separate from the slot. In steps 8–10, the component is no longer compressed and returns to its natural length.

### 2.2. Biomechanical Experiment

Ten subjects without diseases of the musculoskeletal and nervous systems, and without discernible gait abnormalities, were recruited for the experiment. They are aged between 22 and 24 years and have a height range of 160 to 181 cm. Before starting, the objectives and procedures of the experiment are explained to the subjects. This study was reviewed and approved by the research ethics committee of National Taiwan University. The sensors of the wireless Trigno Wireless Biofeedback System, which combines the EMG measurement and an inertial measurement unit (IMU), are attached to the rectus femoris, biceps femoris, tibialis anterior, and gastrocnemius muscles of the subjects’ right lower limbs. Given that the EMG signals are normalized by the maximum voluntary contraction (MVC) signals in this study, the MVC signals were measured before testing. Subsequently, the participants were instructed to perform ten trials of walking at a natural speed, including wearing exoskeletons with three different resilient components and walking without wearing an exoskeleton. Additionally, the squatting and standing-up-from-squatting motions were examined for ten trials for each subject. Prior to each trial, the subjects were permitted to take breaks for relaxation. The EMG signals and angular velocity were recorded and analyzed for the leg with and without the exoskeleton. The statistical software SPSS v22.0 was then employed to compute the normalized (0 to 1) Euclidean distances between curves pairwise.

## 3. Results and Discussion

The EMG and IMU signals for the subjects with and without exoskeleton during level walking and squatting/standing motion are measured and shown in this section. The distinctions between wearing an exoskeleton with various resilient components and not wearing the exoskeleton are also assessed by statistical analysis.

### 3.1. Comparison of EMG Signals

**Figure 4:**
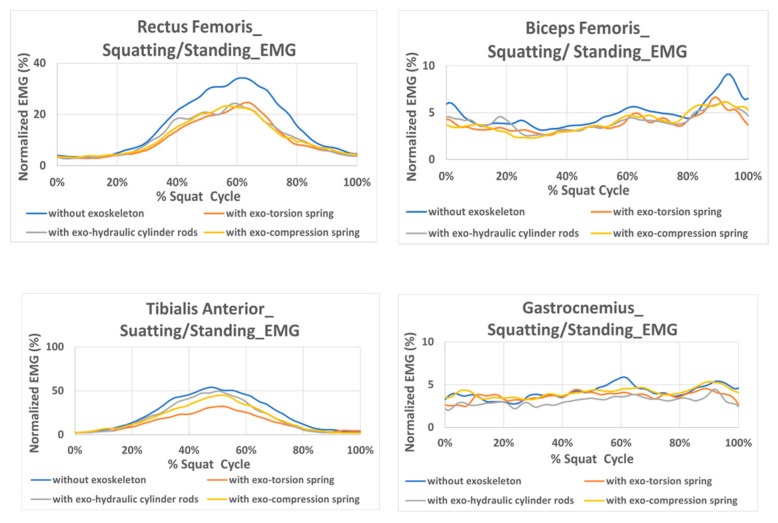
Normalized EMG signal during the squatting/standing motion.

The normalized EMG signals of the ten subjects with/without the exoskeleton during squatting and standing-from-squatting motion are obtained, and the mean values of ten subjects are measured and plotted in Figure 4 for rectus femoris, biceps femoris, tibialis anterior, and gastrocnemius. It is evident that the normalized EMG of the subjects without wearing the exoskeleton is larger than those with the exoskeleton for rectus femoris, biceps femoris, and tibialis anterior, especially for rectus femoris. This indicates that wearing the exoskeleton can reduce muscle effort during squatting/standing. The active muscles align with those involved in the squatting and rising motion, except for the gastrocnemius, which shows unobvious differences between with and without the exoskeleton. It is speculated that the EMG sensor is positioned close to the exoskeleton and could potentially introduce interference during squatting movement.

**Figure 5:**
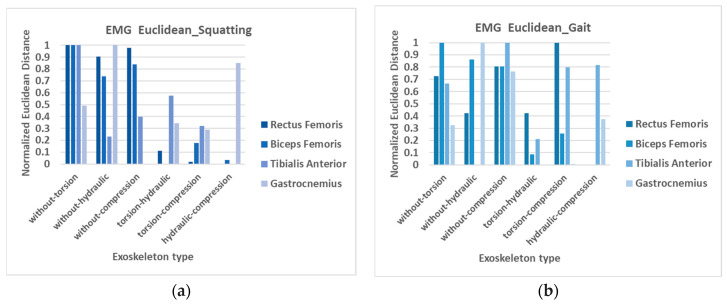
Normalized Euclidean distances between EMG curves (**a**) during squatting/standing, (**b**) during gait.

The normalized Euclidean distances between EMG curves during squatting/standing are shown in Figure 5a. The statistical results showed significant differences between “without exoskeleton” and “with exoskeleton” for the rectus femoris and biceps femoris across all three types of resilient components. This indicates that using this exoskeleton has a significant impact on thigh muscles during squatting/standing movements. The three resilient components show no notable EMG differences during squatting and rising, except for the difference between the hydraulic cylinder rod and the compression spring in the gastrocnemius. Overall, the experimental results indicate that the exoskeleton indeed reduces muscle exertion during squatting and standing movements.

**Figure 6:**
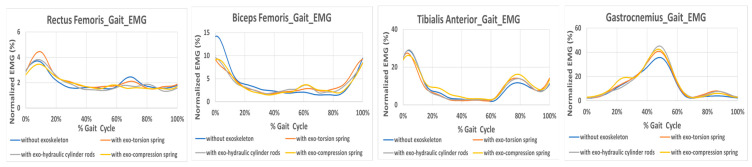
Normalized EMG signal during gait.

In practical applications, the exoskeleton should be designed to be worn all day without the need for repeated donning and doffing. For this purpose, the assistive exoskeleton in this study is designed to offer support during the process of standing up from a squatting position, and the passive power remains inactive for the majority of level walking except during initial and mid-swing phases, to avoid disrupting the natural gait. Additionally, it has been made as lightweight as possible to minimize interference with the user. Nevertheless, it is crucial to investigate the potential impact of the exoskeleton on the body, both when the resilient component is inactive and active. Therefore, the normalized EMG signals during gait with and without the exoskeleton are also obtained for the rectus femoris, biceps femoris, tibialis anterior, and gastrocnemius muscles. The average values of ten subjects are measured and shown in Figure 6. The findings indicate that the muscle effort of the tibialis anterior and gastrocnemius without the exoskeleton is lower during the swing phase compared to when wearing the exoskeleton. This suggests that the exoskeleton results in an increased muscle effort needed during the swing phase to complete the gait motion, particularly for the muscles in the shank. The intended supportive effect during the swing phase, when the knee extends, is not achieved according to the experimental results. This might be attributed to the restriction effects and weight of the exoskeleton. Although the resilient component remains inactive during terminal stance and pre-swing (40–60% of the gait cycle), a higher peak in gastrocnemius activity is observed when wearing the exoskeleton compared to without it during this period. The misalignment between the exoskeleton and the limbs, along with the unnatural movement it causes, may result in increased muscle effort.

Regarding the thigh muscle results, the rectus femoris force during 60–80% of the gait cycle is relatively lower when wearing the exoskeleton. Meanwhile, a slightly increased force exerted by the biceps femoris during 60–80% of the gait while wearing the exoskeleton is observed. The reduced activity of the rectus femoris when wearing an exoskeleton may be due to the assistance provided by the resilient component, but it may also be attributed to compensatory actions by the hamstring muscles or shank muscles. The detailed reasons still require further investigation through more experiments.

It is also interesting to note, as indicated in Figure 6, that the biceps femoris muscle effort at the initial contact of gait without the exoskeleton is greater than that of wearing the exoskeleton. This phenomenon begins from the terminal swing. The greater range of motion without the exoskeleton could lead to this effect, requiring increased effort from the biceps femoris to maintain thigh stability during heel strike. As we can observe, the greater values of flexion/extension angular velocity are at terminal swing. However, it could also result from the assistance provided by the resilient components. Some further study is also needed for verification. The EMG difference between wearing the compression-spring exoskeleton and not wearing the exoskeleton is pronounced for all muscles tested during level walking, as shown in Figure 5b. Different variations are observed with other resilient components.

### 3.2. Comparison of Angular Velocity

**Figure 7:**
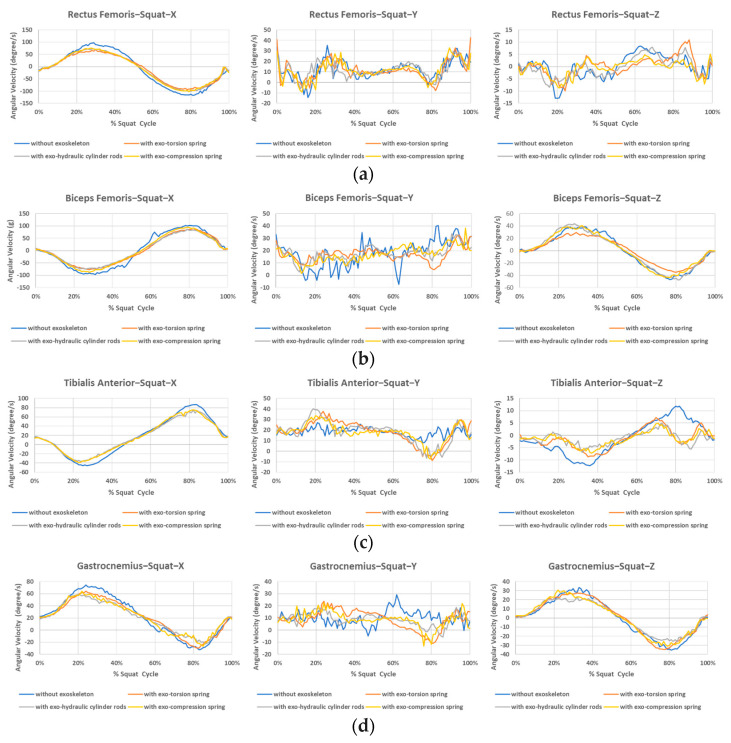
Angular velocity during the squatting/standing motion at different sensor positions. (**a**) Rectus femoris, (**b**) Biceps femoris, (**c**) Tibialis anterior, (**d**) Gastrocnemius.

The angular velocity during squatting/standing motion is obtained by Trigno Wireless Biofeedback System and the average values of ten subjects on x, y and z directions at each sensor position (named by the muscle) are listed in Figure 7a, Figure 7b, Figure 7c and Figure 7d for the position of rectus femoris, biceps femoris, tibialis anterior, and gastrocnemius muscles, respectively. Where x approximately denotes the abduction direction for the front sensors (rectus femoris and tibialis anterior) and adduction for the back sensors (biceps femoris and gastrocnemius muscles), y indicates the downward direction, and z indicates the anterior direction for the front sensors and posterior direction for the back sensors of the right leg when static standing. The rotation about the *x*-axis roughly corresponds to flexion/extension, while rotation about the *y*-axis signifies internal/external rotation, and rotation about the *z*-axis represents abduction/adduction. Due to the sensors being affixed to the skin, the *x*, *y*, and *z* axes do not precisely align with the anatomical axes.

**Figure 8:**
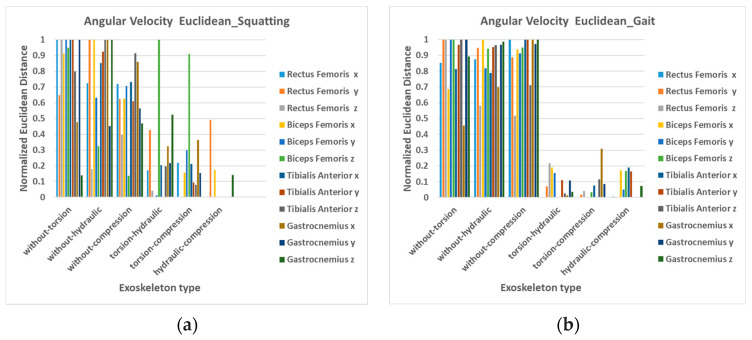
Normalized Euclidean distances between angular velocity curves (**a**) during squatting/standing, (**b**) during gait.

It is evident that the absolute values of angular velocity for flexion/extension motion are greater without the exoskeleton, as shown in the *x*-axis motion of all four sensors in Figure 7. However, this trend is less pronounced in the *z*-axis rotation curves for some muscle positions. Additionally, Figure 8a highlights larger differences between wearing and not wearing the exoskeleton compared to the differences among the three types of exoskeletons.

**Figure 9:**
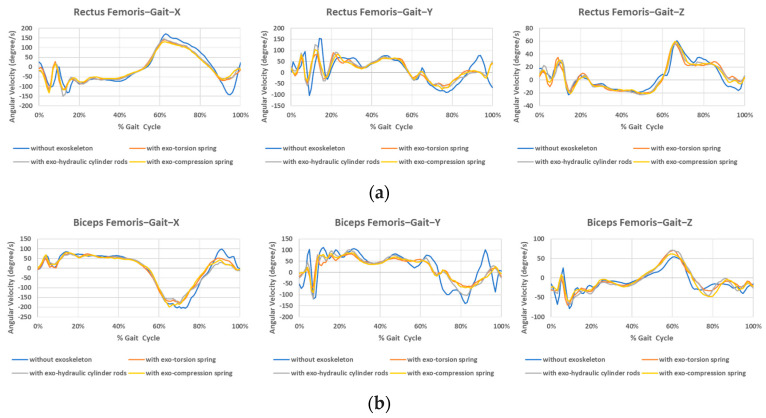

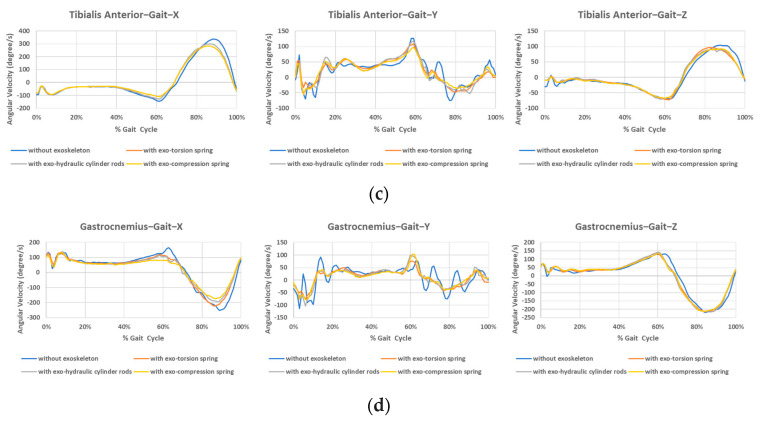
Angular velocity during gait at different sensor positions. (**a**) Rectus femoris, (**b**) Biceps femoris, (**c**) Tibialis anterior, (**d**) Gastrocnemius.

The average angular velocity values of ten subjects during gait at each sensor position are listed in Figure 9. The absolute values of the angular velocity of flexion/extension during the swing phase with all three exoskeletons are significantly lower than those without the exoskeleton, as depicted in Figure 9 (*x*-direction). This suggests that the exoskeleton disrupts the motion of level walking when the resilient component is engaged. An early change in the rotation direction of the thigh is observed in the rectus femoris sensor (occurring within 80–90% of the gait cycle for hip extension), while the tibialis anterior sensor displays an early change in the rotation direction of the shank (around 70% of the gait cycle for knee extension) when wearing the exoskeleton. This suggests that the resilience component may potentially generate a restriction or minor rebound force, causing the leg to straighten in an abnormal manner.

It is also worth noting that the variation of angular velocity for the leg without an exoskeleton is relatively large in the *y*-axis rotation. The waveform oscillation of angular velocity in *y*-axis rotation without exoskeleton shows that wearing the exoskeleton can lead not only to a restriction effect but also to a stabilized effect in rotation motion along this direction. This greater variation in angular velocity could also be attributed to the relative movement of the skin to the bones when not wearing the exoskeleton.

The angular velocity differences between walking without an exoskeleton and walking with an exoskeleton are larger for all three types of exoskeletons, compared to the differences between wearing the three types of exoskeletons, as shown in Figure 8b. This trend is even more pronounced than the angular velocity differences observed during squatting/standing, and the EMG differences of gait. It suggests that when wearing this passive exoskeleton during level walking, the impact on angular velocity is more pronounced than on muscle force. The weight, structure, and range of intervention of the exoskeleton’s resilient components may contribute to the influencing factors, necessitating further research in the future.

## 4. Conclusions

An assistive exoskeleton with a passive powered resilient component has been designed for squatting/standing and gait assistance to assess the exoskeleton’s impact on motion interference. In this study, the resilient component is set to activate during the initial and mid-swing to provide a supporting moment for knee extension without interfering with other phases of the gait. The exoskeleton also provides assistance during the transition from squatting to standing. To elucidate the impact of unnatural motion and misalignment of the exoskeleton on human movement, EMG signals and angular velocity during both squatting/standing and gait are measured. The EMG results indicate that the designed assistive exoskeleton can effectively reduce muscle effort during squatting/standing motion, as intended. However, the wearing of an exoskeleton results in an increase in the shank muscles in the swing phase of gait. The intended supportive effect during the swing phase, when the knee extends, is not effectively achieved. The results from the IMU indicate that wearing the exoskeleton does indeed introduce interference in both squatting/standing and gait motion, even with reduced muscle strength when squatting/standing. Wearing the exoskeleton reduces the absolute values of flexion/extension (*x*-axis) angular velocity during both squatting/standing and the swinging phase of gait. The variation of angular velocity about the *y*-axis during gait is comparatively larger without the exoskeleton compared to when wearing it, suggesting that the exoskeleton may introduce interference but also a stabilizing effect on internal/external rotation during gait. The impact of utilizing various passive powered components in the exoskeleton, including helical compression spring, hydraulic cylinder rod, and torsion spring, is also being compared. The distinctions among the three types of resilient components are insignificant in both squatting/standing and gait motions within this experiment. The statistical results indicate that the differences between not wearing and wearing an exoskeleton are mostly larger than those between wearing three types of exoskeletons for EMG during squatting/standing, angular velocity during gait, and angular velocity during squatting/standing. This trend is particularly evident in the angular velocity during gait, indicating that, in terms of angular velocity, this type of exoskeleton interfered with gait motion, especially in the flexion/extension motion when the resilient component is engaged.

This study enhances our understanding of the kinematic and kinetic effects of wearing a passive exoskeleton during squatting/standing and gait, both with and without the activation of passive powered components. Furthermore, it provides insights into the impact of wearing an exoskeleton throughout the day in real-life scenarios. Future investigations are required to examine the impacts of exoskeleton dimensions, wearing method (alignment), and the inactive duration of the exoskeleton’s resilience component on human motion. This will contribute to the foundation for ongoing advancements in the design of both passive and active powered exoskeletons.

## Data Availability

The original contributions presented in the study are included in the article, further inquiries can be directed to the corresponding author.
